# Synthesis of Ni-Modified ZSM-5 Zeolites and Their Catalytic Performance in *n*-Octane Hydroconversion

**DOI:** 10.3389/fchem.2020.586445

**Published:** 2020-12-11

**Authors:** Qiang Wei, Pengfei Zhang, Xiaodong Liu, Wenbin Huang, Xiayun Fan, Yitong Yan, Rongxun Zhang, Lin Wang, Yasong Zhou

**Affiliations:** State Key Laboratory of Heavy Oil Processing, China University of Petroleum, Beijing, China

**Keywords:** nickel modification, ZSM-5, N-octane, hydroconversion, catalyst

## Abstract

Ni-modified ZSM-5 zeolites with different nickel contents were successfully prepared by the *in situ* synthesis method and the impregnation method. The synthesized samples were characterized by XRD, SEM, N_2_ adsorption–desorption isothermals, and Py-FTIR. The characterization results show that both the textural properties and crystallization of Ni-modified ZSM-5 zeolites were preserved well, and their acidic properties can be modulated after nickel modification. The corresponding NiMo catalysts supported on Ni-modified ZSM-5 zeolites were prepared by the incipient wetness co-impregnation method, and their catalytic performances were evaluated in *n*-octane hydroconversion. Compared to the those modified by the *in situ* synthesis method, ZSM-5 zeolite-supported catalysts modified by the impregnation method exhibit higher stability and higher isomerization selectivity. This is due to the synergistic effect between Brønsted acid sites and Lewis acid sites on the Ni-modified ZSM-5 zeolites, especially for the NiMo/1Ni-Z5 catalyst.

## Introduction

Hydrocracking and hydroisomerization are considered crucial processes in the petroleum refining industry, because they could provide a broad range of high-quality chemicals, gasoline, diesel, and petrol. Long-chain alkanes can be converted into light isomers of alkanes and aromatic products with a high octane number through the hydroconversion catalytic process (Kuznetsov, 2003; Zhou et al., 2008; Sadrameli, 2015). Bifunctional catalysts with metallic and acidic functions are widely used in hydrocracking and hydroisomerization processes. And hydrocracking and hydroisomerization of hydrocarbons over bifunctional catalysts involve rather complex reactions, including (de)hydrogenation, hydrogenolysis, isomerization, aromatization, and cyclization of hydrocarbons (Lugstein et al., 1999). On the one hand, the role of metallic components, like Pt, Pd, Ni, Co, Mo, and their sulfides, is to catalyze hydrogenation and dehydrogenation reactions of hydrocarbons. On the other hand, acidic components over bifunctional catalysts, like Y zeolite, ZSM-5, and zeolite-like solid acids, which possess Brønsted acid sites (BAS), can crack and isomerize olefinic intermediates into smaller olefins (Kuznetsov, 2003). Among the solid acid catalysts, due to its high thermal and hydrothermal stability, ZSM-5 is widely used for isomerization, alkylation, and aromatization of hydrocarbons in the petrochemical industry (Chen et al., 2007; Yamaguchi et al., 2014; Sun et al., 2020). And compared to H-MOR, USY, and H-BEA, ZSM-5 exhibits high selectivity in certain reactions and has attracted much attention in the application as a shape-selectivity catalyst. However, the application of ZSM-5 is restricted because of its microcellular structure, i.e., straight circular channels (5.4 × 5.4 Å) interconnecting with sinusoidal and elliptical channels (5.1 × 5.1 Å) (Wang et al., 2007; Urata et al., 2014). The long diffusion pathway of large molecules, especially for *n*-octane, which is the representative of the alkanes of an industrial hydrocracking feed, would increase residence times of olefinic intermediates and generate carbon depositions in the micro-channels and thus result in the deactivation of catalysts (Pérez-Ramírez et al., 2008; Urata et al., 2014).

In order to enhance activity and suppress carbon deposition, element or compound modifications have attracted much attention (Xue et al., 2009; Rahimi and Karimzadeh, 2011; Kim et al., 2015; Kubo et al., 2015; Tempelman and Hensen, 2015; Zhang et al., 2017). The acid sites on the external surface can be selectively removed via HNO_3_ treatment, and thus, carbon deposition would be inhibited (Inagaki et al., 2013). Phosphorus modification can increase the activity of zeolites (Takahashi et al., 2005; Zhao et al., 2007; Hodala et al., 2014). This can be interpreted as follows. On the one hand, it can stabilize Al in the framework of zeolite and thus enhance its hydrothermal stability; on the other hand, it can decrease the amount of strong acid sites and thus inhibit the overreaction of primary products (Blasco et al., 2006). After phosphorus and lanthanum co-modification, the acidity and basicity of ZSM-5 zeolite can be optimized, and the deposition of carbon would be restrained, and thus, activity and stability can be improved (Furumoto et al., 2012). Similarly, after iron and phosphorus co-modification, the selectivity to propylene of ZSM-5 in catalytic cracking of 1-butene can be improved. Prasanth et al. (2008) found that zeolites after nickel modification can improve the chemical adsorption of hydrogen and be beneficial to the hydrogenation of olefin and thus inhibit overcracking of olefinic intermediates. Other experimental and theoretical results also showed that the incorporation of Ni atom would accelerate the process of H-addition and thus avoid overcracking (Schachtl et al., 2017; Liu et al., 2020a,b; Liu et al., 2021).

In the present work, in order to introduce nickel into the micro-channels of ZSM-5 zeolites, Ni-modified ZSM-5 zeolites were prepared by two different methods. One is that the synthesized ZSM-5 zeolites, which are prepared from the method reported in the literature, were impregnated with excessive NiNO_3_ aqueous solution, namely, the impregnation method. In addition, Ni-modified ZSM-5 zeolites were also synthesized by the *in situ* synthesized method. After that, the effect of different methods on the acidic and textural properties of ZSM-5 zeolites was investigated. Finally, the catalytic performance of Ni-modified ZSM-5 zeolite-supported NiMoS catalysts was evaluated in *n*-octane cracking reaction.

## Experimental

### Reagents

Sodium hydroxide (NaOH, ≥99.8%), aluminum sulfate [Al_2_(SO_4_)_3_, ≥99.8%], nickel nitrate [Ni(NO_3_)_2_, ≥99.8%], and ammonium metatungstate [(NH_4_)_6_W_12_O_39_, ≥99.8%] were purchased from Beijing Modern Oriental Chemical Co. Ltd. Tetraethyl orthosilicate (TEOS, ≥99.8%) and tetrapropylammonium bromide (TPABr, ≥99.8%) were purchased from Guangfu Fine Chemical Co. Ltd.

### Synthesis of ZSM-5 Zeolite

ZSM-5 zeolite with a SiO_2_/Al_2_O_3_ ratio of about 80 was synthesized by a hydrothermal method. In a typical run, mixture A was made by adding tetraethyl orthosilicate (TEOS) into the NaOH aqueous solution; the solution was stirred overnight at ambient temperature for the hydrogenolysis of TEOS. Mixture B was made by dissolving Al_2_(SO_4_)_3_ in an aqueous solution of TPABr and H_2_SO_4_. After stirring for 15 min, mixture B was added dropwise into mixture A. The obtained reaction mixture was stirred at ambient temperature for another 2 h and transferred in a Teflon-lined autoclave for crystallization at 100°C for 24 h and then placed at 160°C for 48 h. After completion of the crystallization, the solid samples were cooled to ambient temperature, washed with distilled water, and dried at 100°C for 12 h. The as-synthesized zeolite was calcined in air for 6 h to remove the templates. ZSM-5 zeolite without modification was denoted as “Z5.”

### Synthesis of Ni-Modified ZSM-5 Zeolite

First, a Ni-modified ZSM-5 zeolite was synthesized by the *in situ* synthesized method. In a typical run, mixture A was made by adding TEOS into a NaOH aqueous solution; the solution was stirred overnight at ambient temperature for the hydrogenolysis of TEOS. Then, appropriate amounts of Ni(NO_3_)_2_ and Al_2_(SO_4_)_3_ were dissolved in an aqueous solution of TPABr and H_2_SO_4_. After stirring for 15 min, mixture B was added dropwise into mixture A. And the following synthesis steps are the same as the preparation procedures of the ZSM-5 zeolite. ZSM-5 zeolite modification by the *in situ* synthesized method was denoted as “xNiZ5,” where “x” represents the mass fraction of nickel.

Second, Ni-modified ZSM-5 zeolite was synthesized by the impregnation method. The obtained ZSM-5 zeolite was impregnated with an excessive aqueous solution which contains the appropriated mass fraction of nickel. ZSM-5 zeolite modification by the impregnation method was denoted as “xNi-Z5,” where “x” represents the mass fraction of nickel.

### Synthesis of NiMo-Supported Catalysts

The supports were obtained by extruding the mixture of 70% of the above-mentioned Ni-modified ZSM-5 zeolites and 30% alumina. The NiMo catalysts were prepared by the incipient wetness co-impregnation method with an aqueous solution of appropriate amount of nickel nitrate and ammonium metatungstate. After impregnation, the prepared samples were aged overnight at ambient temperature, then dried at 120°C for 6 h, and finally calcined at 500°C for 4 h. The corresponding catalysts were denoted as NiMo/Z5, NiMo/xNiZ5, and NiMo/xNi-Z5, respectively.

### Characterization of Materials

Powder X-ray diffraction (XRD) patterns of the obtained ZSM-5 zeolite and nickel-modified ZSM-5 zeolites were carefully analyzed on a PANalyrical advance powder diffractometer in the 2θ range of 5–35° with an interval of 0.1°. Nitrogen adsorption–desorption measurements were performed at 77 K using a Micromeritics ASAP 2010 analyzer on degassed samples (10^−1^ mbar, 573 K, 4 h). The surface area of the examined samples was determined using the Brunauer–Emmett–Teller (BET) equation, and the pore volume and pore diameter were obtained by using the Barrett–Joyner–Halenda (BJH) method with the N_2_ adsorption isotherm. The crystallite size of the synthesized zeolite was observed by field emission scanning electron microscopy (SEM) on a Quanta 200F instrument, and the crystallite size was statistically analyzed by counting at least 500 crystals. Pyridine-absorbed FTIR was characterized on a Bruker Tensor 37 FTIR spectrometer. Pyridine-absorbed FTIR was determined after absorbing and desorbing pyridine at 473 and 623 K, respectively.

### Catalytic Evaluation

The catalytic performance of NiMo catalysts supported on ZSM-5 zeolites with different nickel modifications was assessed in a fixed-bed reactor from 275 to 305°C under H_2_ at 4.0 MPa, and the weight hourly space velocity (WHSV) was fixed at 4 h^−1^. Prior to the assessment, the catalyst was sulfided for 4 h *in situ* using a cyclohexane solution containing 2.0 vol.% CS_2_ at 360°C. The liquid products were collected carefully, and the analysis was performed immediately on an SP-3420 gas chromatograph equipped with a 30-m capillary PONA column. And the column temperature was programmed from 40 to 120°C with a heating rate of 1.5°C/min.

Conversion (X), yield (Yi), and selectivity (Si) of product *i* are calculated according to the following equations (Sun et al., 2020):

$$\begin{array}{l} \text{X}=\left(\text{C in all compounds except octane/C in all compounds in the products}\right)\times 100\%\\ \text{Yi}=\left(\text{Ci/C in all compounds in the products}\right)\times 100\%\\ \text{Si}=\left(\text{Yi/X}\right)\times 100\% \end{array}$$

## Results and Discussion

**Figure 1:**
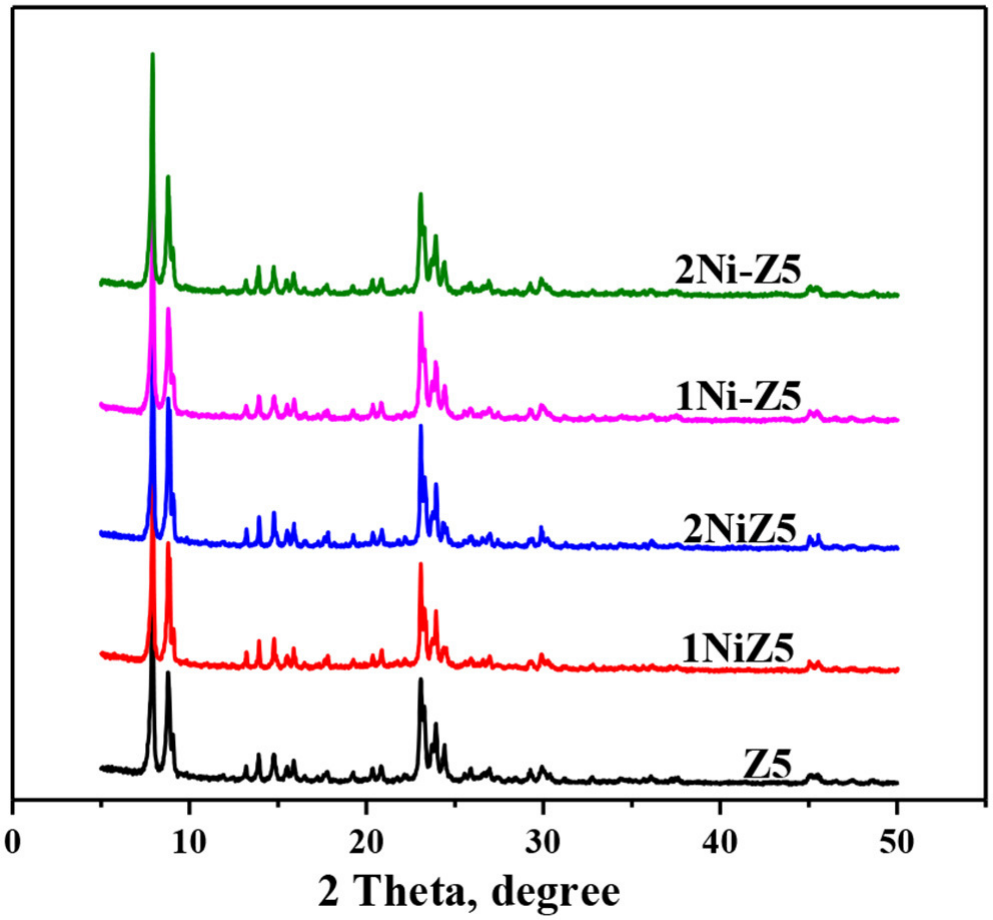
XRD patterns of Ni-modified ZSM-5 zeolites.

**Figure 2:**
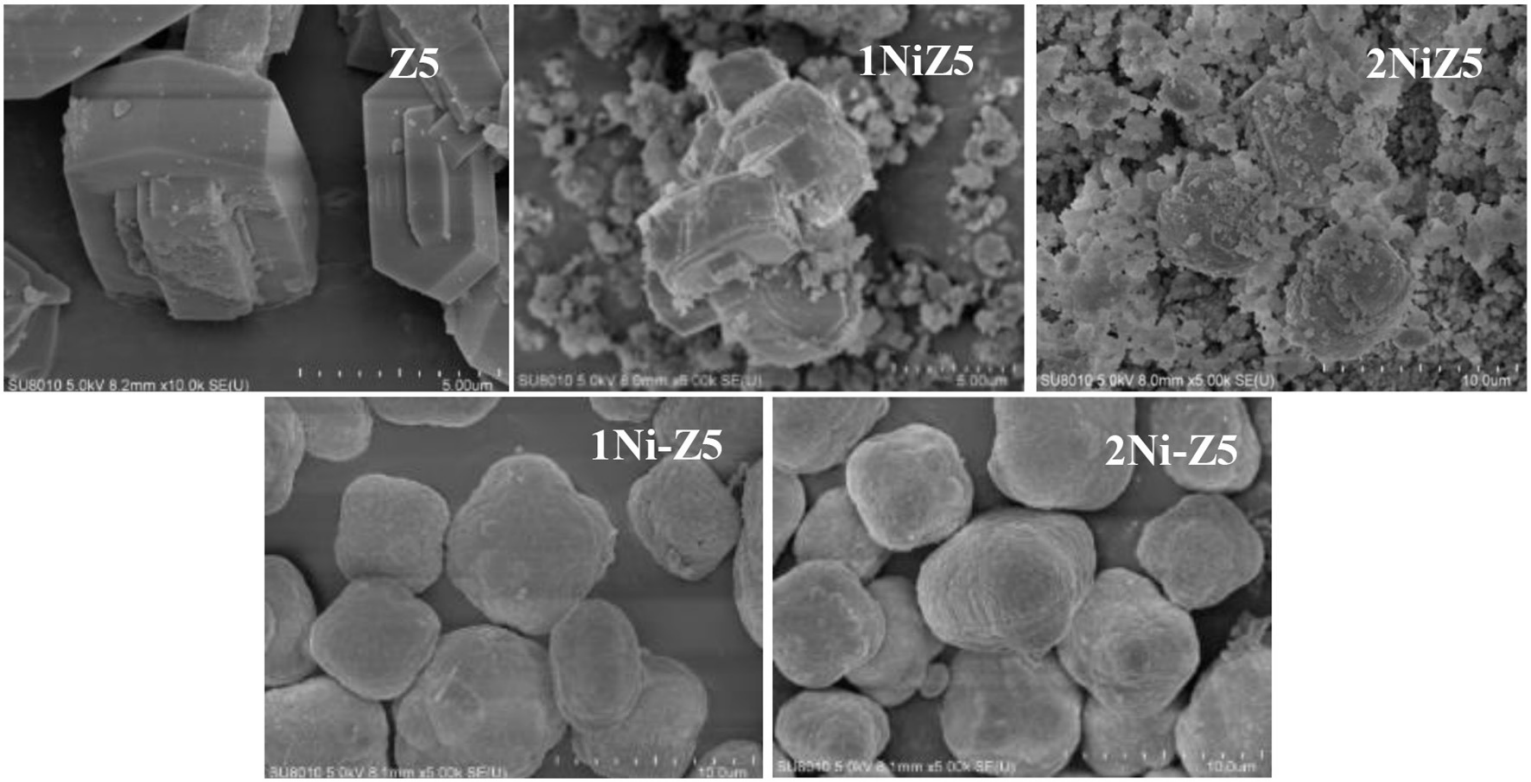
SEM images of Ni-modified ZSM-5 zeolites.

Figure 1 presents the XRD patterns of the parent and Ni-modified ZSM-5 zeolites. It is clearly seen that the characteristic diffraction peaks of all the Ni-modified ZSM-5 zeolites at the 2θ range of 7–9° and 23–25° were maintained completely, indicating that the zeolite units were preserved well after the incorporation of nickel by two different methods. Besides, no other phases related with Ni species were observed, indicating the high dispersion of NiO species introduced by the impregnation method on the surface of ZSM-5 zeolites and the existence of framework Ni species on the ZSM-5 zeolites modified by the *in situ* synthesis method. This is because Al species can be replaced by Ni species in the zeolite framework during the crystallization process (Zhang et al., 2016; Zhou et al., 2017a). Furthermore, the intensities of the typical peaks have no apparent variation after nickel modification. Figure 2 displays the representative SEM images of the Ni-modified ZSM-5 zeolites. The typical morphology of MFI crystals, a uniform hexagonal prism, was observed on the parent ZSM-5 zeolite. The morphology of ZSM-5 zeolites after nickel modification by the *in situ* synthesis method remains unchanged, while the size of ZSM-5 zeolites decreased.

**Figure 3:**
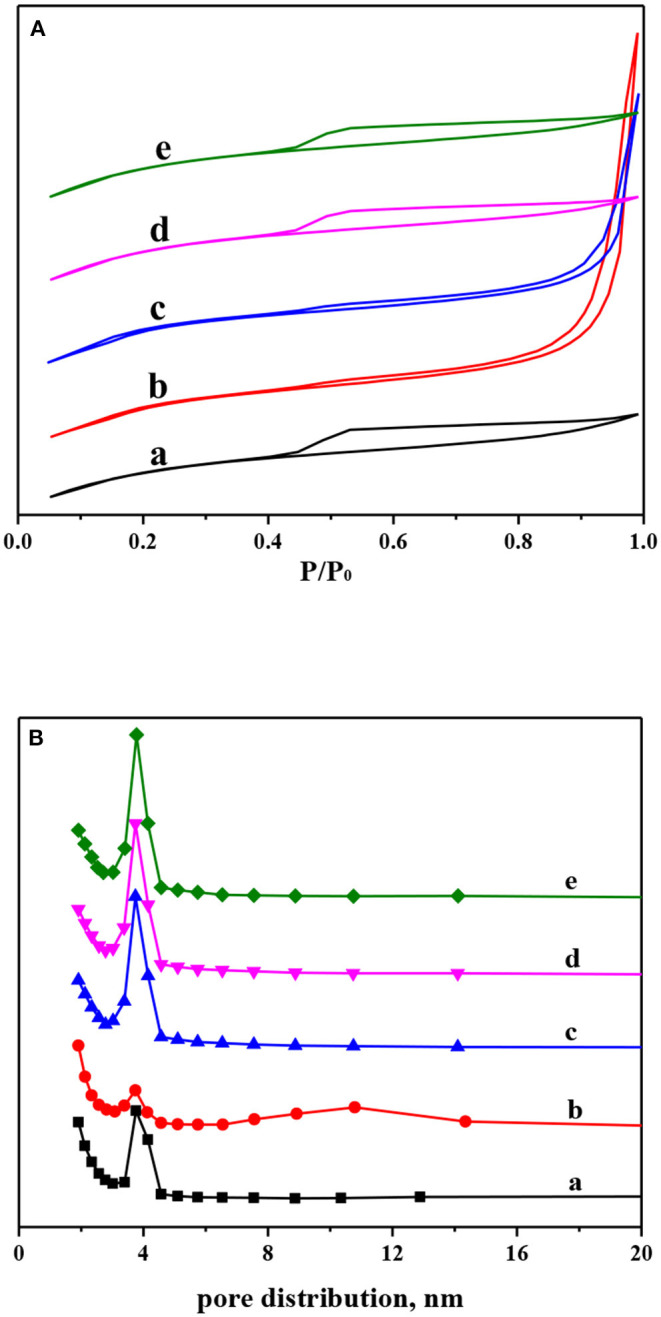
N_2_ adsorption–desorption isotherms **(A)** and BJH pore distribution **(B)** of Ni-modified ZSM-5 zeolites: (a) Z5, (b) 1NiZ5, (c) 2NiZ5, (d) 1Ni-Z5, and (e) 2Ni-Z5.

The N_2_ adsorption–desorption isotherms and pore distribution of Ni-modified ZSM-5 zeolites are shown in Figure 3. And the corresponding BET surface area, pore volume, and pore diameter are displayed in Table 1. As shown in Figure 3A, the parent and Ni-modified ZSM-5 zeolites by the impregnation method have very similar N_2_ adsorption–desorption isotherms. According to the International Union of Pure and Applied Chemistry (IUPAC) classification, the isotherms of those can be attributed to type IV, and the adsorption and desorption branches almost overlapped at relatively low pressure, indicating that the mesoporous structure and the microporous structure were preserved well after nickel modification by the impregnation method (Qi and Yang, 2005; Valencia and Klimova, 2013). Compared to the parent ZSM-5 zeolite, incorporation of small amount of nickel by the impregnation method did not change the BET surface area, pore volume, and pore diameter of those materials (Table 2). After the incorporation of nickel by the *in situ* synthesis method, especially for the 1NiZ5 sample, the isotherms exhibited an obvious hysteresis hoop at high pressure, which originated from the mesoporous structure. This confirmed that fewer microporous structures were destroyed and fewer mesoporous structures were created after the introduced framework Ni species. This is in good agreement with the pore distribution shown in Figure 3B.

**Table 1:** Textural properties of Ni-modified ZSM-5 zeolites with different Ni contents.

**Ni/wt.%**	**D/nm**	**Surface area/(m^2^·g^−1^)**			**V/(cm^3^·g^**−1**^)**
		**BET**	**External**	**Micro**	
Z5	2.12	354	108	245	0.19
1NiZ5	3.54	292	129	163	0.26
2NiZ5	2.21	345	115	230	0.19
1Ni-Z5	2.22	339	111	228	0.19
2Ni-Z5	2.23	343	113	230	0.19

**Table 2:** Acidity properties of Ni-modified ZSM-5 zeolites with different Ni contents.

**Samples**	**Acidity (μmol·g**^****−1****^**)**				
	**Weak acid sites**		**Strong acid sites**		**Total**
	**LAS**	**BAS**	**LAS**	**BAS**	
Z5	13.85	107.94	8.72	85.54	216.05
1NiZ5	12.20	48.47	9.15	38.37	108.19
2NiZ5	12.20	43.08	9.15	33.66	98.81
1Ni-Z5	62.00	72.15	42.50	56.26	232.91
2Ni-Z5	66.00	49.64	45.50	41.70	202.84

**Figure 4:**
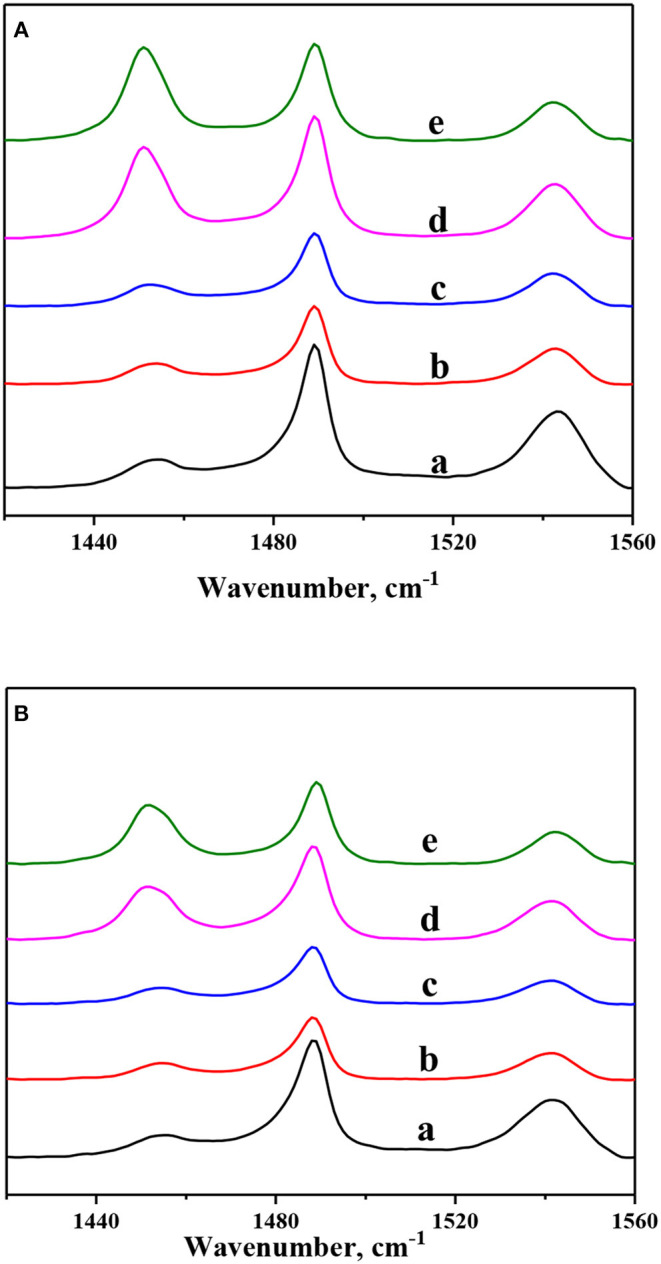
Py-FTIR spectra of Ni-modified ZSM-5 zeolites: (a) Z5, (b) 1NiZ5, (c) 2NiZ5, (d) 1Ni-Z5, and (e) 2Ni-Z5 after desorption at **(A)** at 200°C and **(B)** at 350°C.

In order to investigate nickel modification on the acidity properties of ZSM-5 zeolites, pyridine-adsorbed FTIR spectra of Ni-modified ZSM-5 zeolites are recorded at different temperatures and exhibited in Figure 4. And the amounts of BAS and Lewis acid sites (LAS) are calculated and shown in Table 3. According to the literature (Pawelec et al., 2008; Gutiérrez et al., 2014), the vibration peak centered at ~1,450 and 1,540 cm^−1^, which can be ascribed to the pyridine ions adsorbed on the LAS and BAS, respectively. As shown in Figure 4, because the parent ZSM-5 zeolite had the highest crystallinity, it exhibited the highest amount of BAS (weak, 107.94 μmol·g^−1^; strong, 85.54 μmol g^−1^) but the lowest LAS (weak, 13.85 μmol·g^−1^; strong, 8.72 μmol·g^−1^). After the introduction of 1 wt.% nickel by the *in situ* synthesis method, the amount of BAS (the amount of both weak and strong acid sites) shows a dramatic decrease (from 193.48 to 86.84 μmol·g^−1^), which can be attributed to the decrease of Si/Al. And with further nickel introduction (2 wt.%), the amount of BAS shows a smaller decrease (from 86.84 to 76.74 μmol·g^−1^). On the other hand, the amount of LAS (both the weak and strong acid sites) was almost unchanged. The amount of BAS (both the weak and strong acid sites) also decreased after nickel modification by the impregnation method. Compared to that after nickel modification by the *in situ* synthesis method, BAS decreased by a smaller amount after nickel modification by the impregnation method. However, the amount of LAS (both the weak and strong acid sites) increased sharply after the introduction of 1 wt.% nickel by the impregnation method (from 22.57 to 104.5 μmol·g^−1^) and increased slightly with the further introduction of nickel to 2 wt.% (from 104.5 to 111.5 μmol·g^−1^). This result can be explained by the fact that BAS are only generated from framework Al species in the zeolites; the impregnation solution of nickel nitrate was acidic, so the process of impregnation led to dealumination from ZSM-5 zeolite frameworks and thus decreased the BAS (Fricke et al., 2000; Zhou et al., 2017b). Furthermore, some LAS can be formatted in the NiO granular (Zhou et al., 2017a). Therefore, the existence of no-framework Al species and Ni species can increase the amount of LAS.

**Table 3:** Product selectivity of *n*-octane hydroconversion.

	**NiMo/1Ni-Z5**	**NiMo/2Ni-Z5**	**NiMo/Z5**	**NiMo/1NiZ5**	**NiMo/2NiZ5**
C1–C3	0.35	1.09	1.74	2.59	4.00
iso-C4	8.90	9.55	10.63	11.17	11.51
*n*-C4	23.25	24.78	20.87	21.06	20.23
iso-C5	23.56	19.87	16.11	13.45	11.27
*n*-C5	12.97	16.85	16.37	16.50	16.82
iso-C6	11.08	10.05	10.64	9.80	9.15
*n*-C6	2.25	3.18	4.98	5.40	6.35
iso-C7	6.84	6.90	6.95	6.60	6.71
*n*-C7	0.54	0.50	0.86	1.60	2.31
iso-C8	9.83	7.10	9.94	10.76	10.84
iso-sum	60.21	53.47	54.27	51.78	49.48

**Figure 5:**
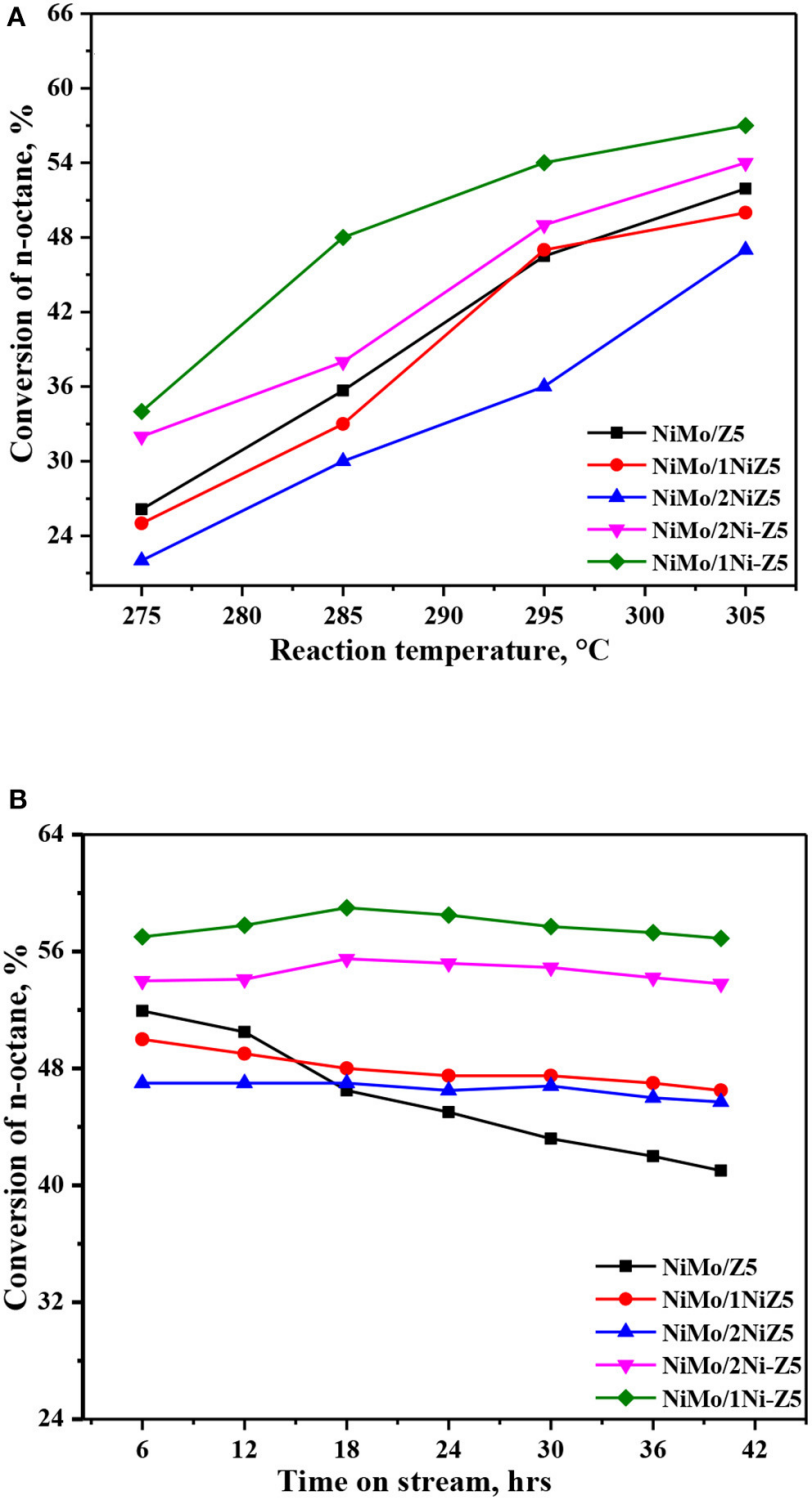
Conversion of *n*-octane at different temperatures **(A)** and conversion with TOS **(B)** over the series catalysts.

The *n*-octane hydroconversion was carried out in order to investigate the effect of nickel modification on the activity and stability of ZSM-5 zeolite-supported catalysts. The effects of the reaction temperature on the conversion of *n*-octane and *n*-octane conversion as a function of time on stream (TOS) are displayed in Figure 5. As presented in Figure 5A, the catalytic ability of this series catalysts decreased in the following order: NiMo/1Ni-Z5 > NiMo/2Ni-Z5 > NiMo/Z5 > NiMo/1NiZ5 > NiMo/2NiZ5. This means that in comparison with the reference catalyst (NiMo/Z5), a remarkable decrease in *n*-octane conversion was observed in NiMo/xNiZ5 catalysts prepared by the *in situ* synthesis method, while a strong increase in *n*-octane conversion was observed on the NiMo/xNi-Z5 catalyst prepared by the impregnation method. It can be found from Figure 5B that after nickel modification by the *in situ* synthesis method or impregnation method, NiMo catalysts supported on Ni-modified ZSM-S zeolites show better stability in *n*-octane hydroconversion than the reference catalyst (NiMo/Z5), with more gradual curves of TOS. Therefore, the results show that the introduction of nickel by the impregnation method on ZSM-S zeolite-supported catalysts can efficiently improve the catalytic activity and stability of *n*-octane hydroconversion.

The product selectivity of *n*-octane over the five catalysts was obtained at ~20% *n*-octane conversion and listed in Table 3. Under the conditions studied, cracking was the main reaction over this series catalyst. It can be seen that olefins and products with a carbon number exceeding 8 were not detected in these experiments. Iso-pentane, *n*-pentane, iso-butane, *n*-butane, and iso-hexane are the major products of *n*-octane hydroconversion. And isomerization selectivity decreased in this order: NiMo/1Ni-Z5 > NiMo/2Ni-Z5 > NiMo/Z5 > NiMo/1NiZ5 > NiMo/2NiZ5.

Although the incorporation of nickel by the *in situ* synthesis method into the framework of ZSM-5 zeolites improved the catalytic stability of NiMo/NiZ5 catalysts, a decrease in *n*-octane conversion and isomerization selectivity were observed. This is because a remarkable decrease of BAS would diminish the rate of cracking and isomerization of *n*-octane when Ni-modified ZSM-5 zeolites were synthesized by the *in situ* synthesis method. Conversely, NiMo/xNi-Z5 catalysts exhibited a high catalytic stability and ability with high isomerization selectivity, which can be attributed to the synergistic effect of BAS and LAS. On one hand, the remarkable increase of the amount of LAS, which originates from no-framework Al species and Ni species, produced more carbonium ion species. Accordingly, the *n*-octane conversion and carbonium ion isomerization reactions would be promoted by the increasing carbonium ion intermediates. On the other hand, the incorporation of nickel into micropores of ZSM-5 zeolites would shorten the distance between BAS and LAS, and thus, the formed carbonium ion species could efficiently transform from LAS to BAS. When the nickel content was 1%, the NiMo/1Ni-Z5 catalyst showed the highest ability with high isomerization selectivity, which can be attributed to the optimal amount of BAS and LAS.

## Conclusions

Ni-modified ZSM-5 zeolites with different nickel contents were prepared by two different methods, namely, *in situ* synthesis and impregnation methods. The XRD and SEM analyses showed that ZSM-5 zeolites were preserved well after the introduction of a small amount of nickel. And BET analysis showed that the textural properties of Ni-modified ZSM-5 zeolites, especially for the microporous surface area, were hardly affected, indicating that those micropores were also not blocked by nickel. But the Py-FTIR characterization showed that the total amount of acid sites, especially for the strong acid sites, decreased by the introduction of nickel to ZSM-5 zeolites.

During *n*-octane hydroconversion, compared with the NiMo/Z5 catalyst, although NiMo/xNiZ5 catalysts exhibit a good stability with gradual curves of TOS, a decrease in *n*-octane conversion and isomerization selectivity was observed, which can be attributed to the remarkable decrease of BAS after nickel modification of ZSM-5 zeolites by the *in situ* synthesis method. Conversely, the results show that NiMo/xNi-Z5 catalysts after nickel modification by the impregnation method can improve the catalytic stability as there are more steady curves of TOS and ability with higher isomerization selectivity. This can be interpreted as follows: the increase of LAS would produce more carbonium ion species, and the formed carbonium ion species can efficiently transform LAS into BAS after the incorporation of nickel into micropores of ZSM-5 by the impregnation method.

## Data Availability Statement

The original contributions presented in the study are included in the article/supplementary material, further inquiries can be directed to the corresponding author/s.

## Author Contributions

QW designed the experiment and carried out data analysis. PZ, XL, WH, XF, YY, RZ, and LW carried out the experiment. YZ designed the experiment. All authors contributed to the article and approved the submitted version.

## Conflict of Interest

The authors declare that the research was conducted in the absence of any commercial or financial relationships that could be construed as a potential conflict of interest.
